# A case of successful salvage despite right ventricular perforation during AVEIR VR leadless pacemaker implantation

**DOI:** 10.1002/joa3.70000

**Published:** 2025-01-23

**Authors:** Masanori Matsuo, Kenji Shimeno, Naoki Matsumoto, Yukio Abe, Daiju Fukuda

**Affiliations:** ^1^ Department of Cardiology Osaka City General Hospital Osaka Japan; ^2^ Department of Cardiovascular Medicine Osaka Metropolitan University Graduate School of Medicine Osaka Japan

**Keywords:** Aveir VR, cardiac perforation, cardiac tamponade, leadless pacemaker, transcatheter pacing system

## Abstract

To prevent cardiac tamponade caused by catheter tip slippage during the retraction of the protective sleeve in Aveir VR implantation, it is crucial to carefully evaluate not only the bulge of the protective sleeve but also the shape of the system's shaft using fluoroscopic imaging.

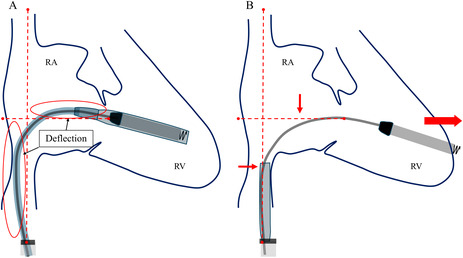

The incidence of cardiac perforation associated with leadless pacemaker implantation in previous studies is low.^1^ However, if it does occur, it often requires intervention and carries a higher risk of mortality compared to transvenous leads.^2^ We report a case of successful salvage despite right ventricular perforation during leadless pacemaker implantation.

The patient was a 91‐year‐old female with a history of hypertension, chronic kidney disease, and lung cancer. Her body weight and her body mass index were 42.3 kg and 19.6 kg/m^2^. We decided to implant a pacemaker because of complete atrioventricular block accompanied by dyspnea on exertion. When presenting the options of a transvenous pacemaker and a leadless pacemaker to the patient and her family, they expressed a preference for the implantation of a leadless pacemaker. She presented with renal dysfunction, as indicated by a serum creatinine level of 2.25 mg/dL and an estimated glomerular filtration rate of 16 mL/min. We wanted to avoid the use of contrast agents in this case. Aveir VR has been reported to require fewer deployment attempts,^3^ which led us to believe that the amount of contrast agent used could be minimized. Based on this reasoning, we chose Aveir VR for this patient.

We initially placed the Aveir introducer in the right femoral vein, followed by the administration of 3000 units of heparin. We positioned a pigtail catheter at the right ventricular apex and used it as a reference to adjust the left anterior oblique (LAO) angle, allowing the ventricular septum to appear more vertical. Following right ventriculography, we inserted the device system into the right ventricle (RV) and injected contrast medium through the protective sleeve irrigation port to ensure that the tip was positioned on the RV septal side (Figure 1, Materials S1 and S2). Subsequently, we unlocked and retracted the protective sleeve. During this maneuver, the device tip advanced, as evidenced by fluoroscopy in the right anterior oblique (RAO) view (Figure 2A,B). A current of injury before device fixation was absent on the commanded electrogram (Figure 2C). Shortly thereafter, the patient's blood pressure dropped, and contrast extravasation into the pericardial space was observed, resulting in the diagnosis of cardiac perforation (Material S3). We immediately reversed heparin and performed percutaneous pericardial drainage while administering norepinephrine. Successful neutralization was confirmed with an activated clotting time of 129 s. Although transient hemodynamic improvement was noted, drainage of blood from the pericardial cavity gradually became progressively more challenging, leading to recurrent hemodynamic instability. Echocardiography suggested that blood coagulation within the pericardial cavity was obstructing effective drainage. Because of the sustained hemodynamic instability, we initiated percutaneous cardiopulmonary support and planned for surgical removal of the device and repair of the RV. In consideration of the potential for further bleeding and worsening hemodynamic status, we refrained from moving the perforating device. Upon opening the pericardium, the device was found protruding from the anterior wall of the RV (Figure 3). The tear in the RV was repaired with several sutures. The patient's postoperative course was uneventful. On postoperative day 21, she underwent transvenous pacemaker implantation and was subsequently discharged.

**FIGURE 1:**
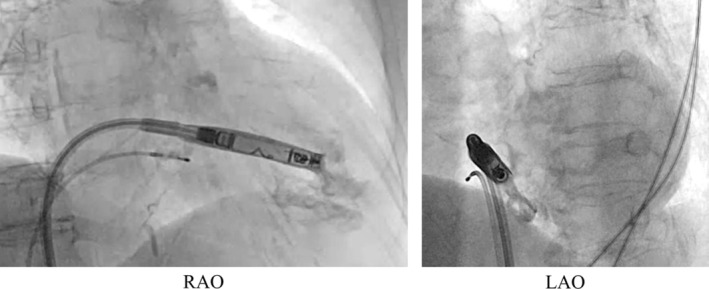
Contrast injection through protective sleeve irrigation port. LAO, left anterior oblique; RAO, right anterior oblique.

**FIGURE 2:**
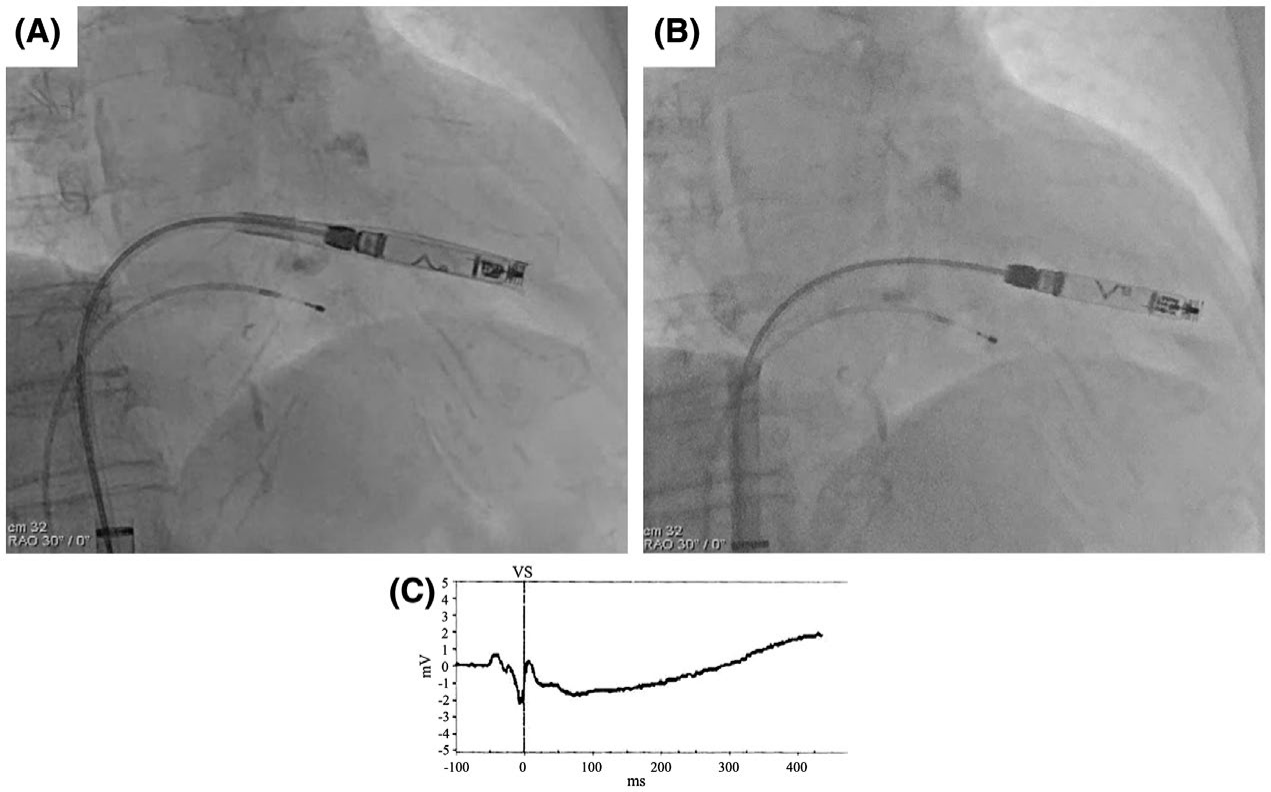
(A) Before the protective sleeve was retracted. (B) After the protective sleeve was retracted. (C) The commanded electrogram before fixation.

**FIGURE 3:**
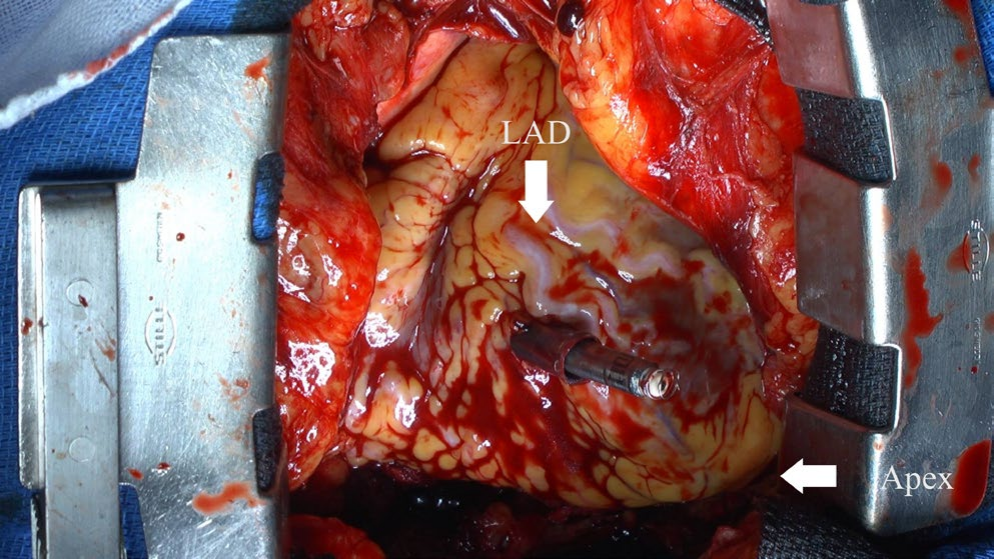
Intraoperative image. LAD, left anterior descending artery.

When comparing the shape of the delivery system's shaft before and after sleeve retraction using RAO fluoroscopic images, the shaft was found to be more flexed before retraction than after. This deflection is thought to indicate a certain degree of compression between the system and the RV. The protective sleeve is stiffer compared to the inner shaft. Therefore, when the protective sleeve is retracted while the shaft is in a deflected state, the entire system attempts to straighten. Consequently, it is hypothesized that this recoil caused the tip to advance forward (Figure 4). During the actual procedure, since there was no evidence of bulging in the protective sleeve, we did not believe that the system was exerting excessive pressure on the RV. However, as we retracted the protective sleeve, the catheter tip unintentionally slipped forward. This case suggests that when retracting the protective sleeve, it is crucial to evaluate not only the shape of the sleeve but also the shape of the system's shaft using RAO and LAO fluoroscopic images, in order to accurately assess whether the catheter tip is exerting pressure on the RV wall. A critical factor that may have contributed to the successful rescue despite the occurrence of perforation was the decision to refrain from the device removal until surgical intervention. The Aveir VR was large, measuring 19.5 Fr and 6.44 mm in diameter, and if we had removed the device before surgery, it could have further deteriorated the patient's condition. Based on our experience in this case, it is advisable that if cardiac tamponade occurs during leadless pacemaker implantation, the device should not be hastily removed.

**FIGURE 4:**
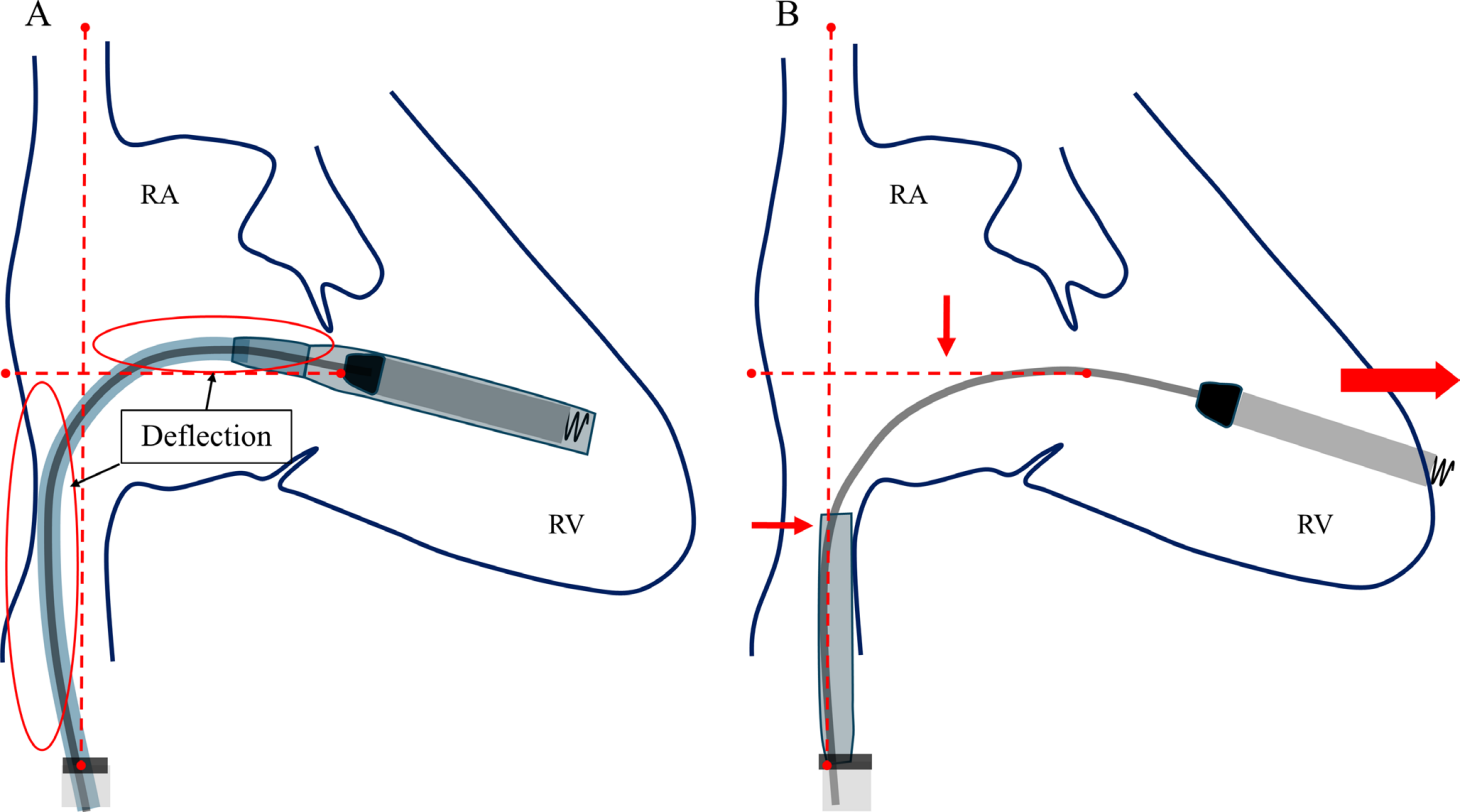
A schematic diagram of RAO fluoroscopic images before and after the protective sleeve retraction.

Among the risk factors for pericardial effusion in Micra (Medtronic, Minneapolis, MN, USA) implantation,^4^ this patient had three risk factors: female, older age, and lower body weight. These factors may suggest myocardial fragility, which is probably a risk factor for Aveir implantation as well. Patients with many risk factors would require more attention because of the potential for cardiac perforation when the catheter tip slips forward. To facilitate safer device implantation, it is imperative to identify the procedural stages during which perforation is most likely to occur, warranting further research in this domain. We present this case with the intention of preventing similar incidents in the future.

## CONFLICT OF INTEREST STATEMENT

The authors declare no conflict of interests for this article.

## ETHICS STATEMENT

Informed consents were obtained from the patients to publish the case report.

## PATIENT CONSENT STATEMENT

Patient consent has been obtained for this report.

## Supporting information

Material S1.

Material S2.

Material S3.

## Data Availability

The data underlying the results are available within the article.
